# Different Strategies to Overcome Resistance to Proteasome Inhibitors—A Summary 20 Years after Their Introduction

**DOI:** 10.3390/ijms25168949

**Published:** 2024-08-16

**Authors:** Paweł Tyrna, Grzegorz Procyk, Łukasz Szeleszczuk, Izabela Młynarczuk-Biały

**Affiliations:** 1 Histology and Embryology Students’ Science Association, Department of Histology and Embryology, Faculty of Medicine, Warsaw Medical University, Chalubinskiego 5, 02-004 Warsaw, Poland; pawel.tyrna@gmail.com; 2 1st Chair and Department of Cardiology, Medical University of Warsaw, Banacha 1A, 02-097 Warsaw, Poland; grzegorz.procyk@wum.edu.pl; 3 Department of Organic and Physical Chemistry, Faculty of Pharmacy, Medical University of Warsaw, Banacha 1 Str., 02-093 Warsaw, Poland; lukasz.szeleszczuk@wum.edu.pl; 4 Department of Histology and Embryology, Faculty of Medicine, Warsaw Medical University, Chalubinskiego 5, 02-004 Warsaw, Poland

**Keywords:** proteasome inhibitors, molecular medicine, treatment resistance

## Abstract

Proteasome inhibitors (PIs), bortezomib, carfilzomib, and ixazomib, are the first-line treatment for multiple myeloma (MM). They inhibit cytosolic protein degradation in cells, which leads to the accumulation of misfolded and malfunctioned proteins in the cytosol and endoplasmic reticulum, resulting in cell death. Despite being a breakthrough in MM therapy, malignant cells develop resistance to PIs via different mechanisms. Understanding these mechanisms drives research toward new anticancer agents to overcome PI resistance. In this review, we summarize the mechanism of action of PIs and how MM cells adapt to these drugs to develop resistance. Finally, we explore these mechanisms to present strategies to interfere with PI resistance. The strategies include new inhibitors of the ubiquitin–proteasome system, drug efflux inhibitors, autophagy disruption, targeting stress response mechanisms, affecting survival and cell cycle regulators, bone marrow microenvironment modulation, and immunotherapy. We list potential pharmacological targets examined in in vitro, in vivo, and clinical studies. Some of these strategies have already provided clinicians with new anti-MM medications, such as panobinostat and selinexor. We hope that further exploration of the subject will broaden the range of therapeutic options and improve patient outcomes.

## 1. Introduction

Protein degradation is one of the mechanisms responsible for maintaining correct cellular functions. Not only does it remove misfolded and damaged proteins, but it also allows for the orchestration of complex intracellular processes, such as cell cycle control. The ubiquitin–proteasome system (UPS) plays a vital role in this process and is responsible for the degradation of most intracellular proteins in eukaryotic cells [1,2].

Inhibition of the UPS was proved to be a successful cancer treatment method. Despite the initial concern about toxicity against healthy cells, inhibitors of the 26S proteasome exhibited selectivity for malignant cells. Currently, three proteasome inhibitors (PIs), bortezomib (BTZ), carfilzomib (CFZ), and ixazomib (IXZ), are registered for the treatment of multiple myeloma (MM) and mantle-cell lymphoma (MCL). During treatment, malignant cells develop resistance to PIs, which leads to disease relapse [3,4].

As our knowledge of resistance mechanisms to PIs broadens, new strategies to overcome them are continuously emerging. Several dozen potential targets whose modulation may overcome resistance to PIs have been described. Advances in this field led to the approval of two new drugs in MM: panobinostat, a histone deacetylase inhibitor, and selinexor, an exportin-1 inhibitor [5,6]. In the coming years, other compounds will likely join them. Immunotherapy emerges as an up-and-coming treatment option. Monoclonal antibodies directed against CD38 or B-cell maturation antigen (BCMA) showed good efficacy in a clinical setting. Even better outcomes were achieved in patients treated with CAR-T cells, a treatment modality with exceptionally high specificity and efficacy.

Resistance to PIs has been the subject of several reviews so far. Niewerth et al. and Wallington-Beddoe et al. elegantly summarized the resistance to PIs, describing various potential mechanisms [4,7]. However, these reviews were published in 2018 and 2014, respectively. Since then, our knowledge has expanded substantially, and many new treatment options have appeared. Regarding more recent attempts to summarize the knowledge about resistance to PIs, a paper from 2023 by Kozalak et al. must be mentioned [8]. The authors present the potential molecular mechanisms of growing resistance but mostly focus on preclinical models and new technologies. Another interesting review in this field was published by Davis et al. [9]. However, resistance to PIs was not exhaustively described in this article as the authors also aimed to discuss resistance to immunomodulatory drugs and monoclonal antibodies. The same limitation applies to the other review in the field, which was written by Pinto et al. [10].

Regarding the above, we identify an unmet need for a comprehensive, up-to-date summary of the existing knowledge about the resistance to all PIs. Therefore, we aimed to thoroughly review and discuss this field’s available literature. The novelty of our review is its comprehensiveness in an unequivocally defined research problem. We present the physiological role of UPS. Then, we depict the role and mechanism of action of PIs, including their chemical structure. Furthermore, we present the developing problem of resistance to PIs. Importantly, we discuss all significant up-to-date strategies to overcome this resistance. Moreover, we present the outcomes of these strategies in a clinical setting. Finally, we analyze the future perspectives and potential directions of development.

## 2. The Ubiquitin–Proteasome System

The UPS is the primary pathway of cytosolic protein degradation in eukaryotic cells. It can be divided into two parts: (i) tagging the target protein for degradation and (ii) proteolysis in the proteasome [1].

### 2.1. Degradation Signal

**Figure 1:**
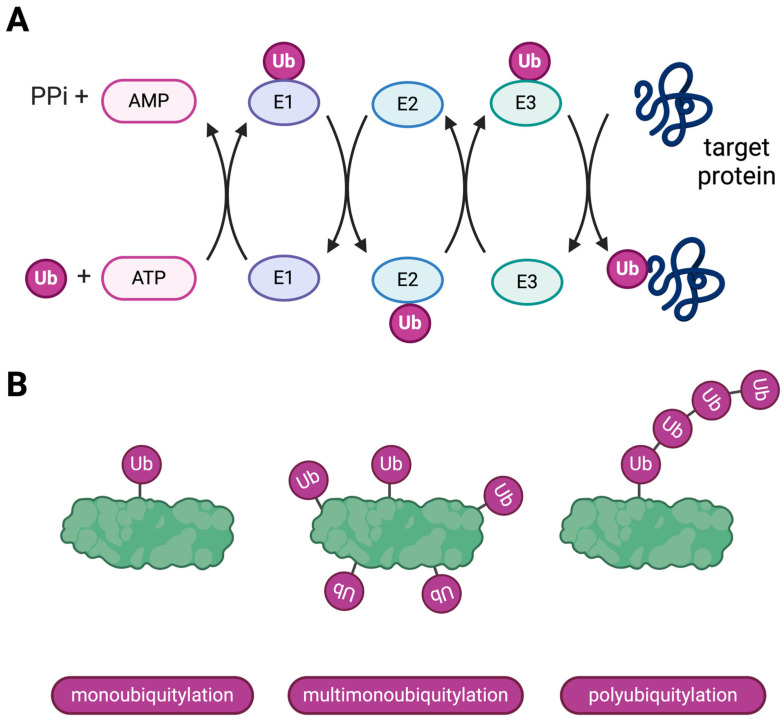
Schematic representation of the ubiquitylation machinery. (**A**). Three consecutive reactions lead to ubiquitin (Ub) attachment to the target protein. First, the E1 enzyme forms a covalent bond with Ub at the expense of energy from ATP. Then, Ub is transferred from the E1 to the E2 enzyme. Finally, the E3 enzyme catalyzes the transfer of Ub from E2 to the target protein. (**B**). Basic notions in the ubiquitylation code. A monoubiquitylated protein has a single Ub molecule attached to it. A multimonoubiquitylated protein has several Ub molecules attached at different sites. A polyubiquitylated protein has several Ub molecules attached as a chain at one site of this protein. This figure was created with BioRender.com.

Most proteins are tagged for degradation by a unique 76-amino acid polypeptide called ubiquitin (Ub). Ub is attached to the target protein by an isopeptide bond. The attachment of Ub to the target protein (ubiquitylation) is performed by three consecutive enzymes: Ub-activating enzyme (E1), Ub-conjugating enzyme (E2), and Ub ligase (E3) (Figure 1A) [2]. In humans, there are at least two types of E1 enzymes, about 40 types of E2 enzymes, and about 600 types of E3 enzymes. This complexity enables precise regulation of selecting proteins for proteolysis [11].

The E1 enzyme forms a covalent thioester bond with Ub, which requires energy from ATP hydrolysis. Then, the E2 enzyme transfers Ub from E1 to itself. The E3 enzyme catalyzes the final transfer from E2 to the target protein [11]. The Ub molecule can be attached either directly to one of the protein’s lysyl residues or to another previously attached Ub molecule. In the formation of a polymeric ubiquitin chain, the C terminus of a given ubiquitin molecule is covalently conjugated to one of seven lysyl residues (K6, K11, K27, K29, K33, K48, or K63) [12]. This diversity allows polyubiquitylation to regulate many cellular events. Therefore, a protein can be tagged with (i) a single Ub molecule (monoubiquitylation), (ii) several Ub molecules at different sites (multimonoubiquitylation), or (iii) several Ub molecules attached one to another as a poly-Ub chain (polyubiquitylation) (Figure 1B) [12]. Poly-Ub chains linked via lysine 48 (K48) are the most abundant and the canonical signal for ATP-dependent proteasomal degradation. Meanwhile, chains linked via lysine 63 (K63) play other roles and might be related to lysosomal degradation [12,13,14]. Some proteins can be degraded in a ubiquitin-independent manner [15].

### 2.2. The Proteasome

**Figure 2:**
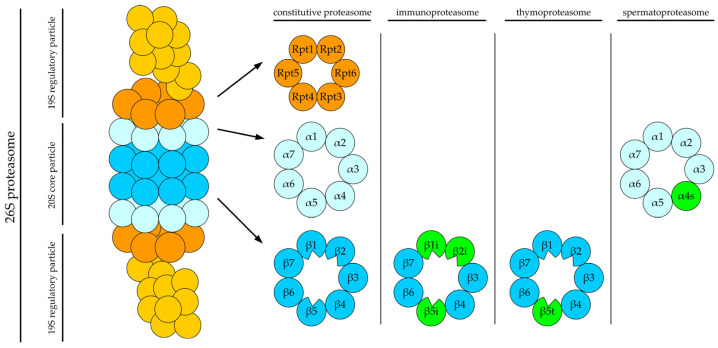
Schematic structure of the constitutive 26S proteasome and its tissue-specific variants. The proteasome comprises the 20S core particle and the 19S regulatory particle at one or both ends of the core particle. The core particle contains four heptameric rings (two inner β rings and two outer α rings) stacked on top of each other. Each inner β ring possesses three catalytic subunits (β1, β2, and β5, all depicted with notches), which cleave the target protein into peptides. The outer α rings gate the way to the interior of the core particle. The 19S regulatory particle is composed of (i) a hexameric ring built of Rpt ATPases (the base) unfolding the target protein and (ii) a multi-subunit complex (the lid) responsible for the recognition of target proteins. Tissue-specific proteasomes contain analogs of selected subunits (green in the figure), altering protein specificity and allowing different peptides to be generated. Immunoproteasomes contain β1i, β2i, and β5i subunits, thymoproteasomes have a β5t subunit, and spermatoproteasomes contain a α4s subunit.

The 26S proteasome is a multi-subunit enzymatic complex present in the nucleus and cytoplasm of eukaryotic cells. It comprises the 20S core and 19S regulatory particles (Figure 2).

The 20S core particle is built of four rings stacked on each other. Each ring is a heptamer. The top and bottom rings contain α subunits, whereas the two middle rings contain β subunits. There is a channel running through the middle of all four rings. The α rings function as a gate, preventing accidental entrance of proteins into the channel, while the β rings perform the actual proteolysis. The β1, β2, and β5 subunits possess the caspase-like, trypsin-like, and chymotrypsin-like activities, respectively [16,17]. Therefore, the entire proteasome contains six active sites exposed in the middle of the channel. The degraded protein passes through the channel to undergo enzymatic decomposition into oligopeptides.

Apart from the typical, constitutive core particle described above, certain types of cells possess specific proteasome variants. The structure of these variants is similar to the constitutive proteasome, but several subunits are replaced by their analogs: β1i, β2i, and β5i in immunoproteasomes, β5t in thymoproteasomes (in the thymus of vertebrae where it is involved in the positive selection process during the maturation of T lymphocytes), and α4s in spermatoproteasomes (in spermatozoa where it is crucial for germ cell development [18]). Replacing the conventional catalytic subunits with their tissue-specific counterparts alters substrate specificity and generates a different set of peptides from the same substrate. This is important for the functioning of the immune system because immuno- and thymoproteasomes generate peptides for antigen presentation [19,20].

On one or both ends of the 20S core particle lies the 19S regulatory particle. The regulatory particle recognizes proteins tagged for lysis and unfolds them into a polypeptide chain, which can enter the core particle for lysis [16]. The regulatory particle is composed of multiple subunits. Some function as Ub receptors, e.g., Rpn2, Rpn10, and Rpn13. The Rpn11 subunit possesses deubiquitinase activity, removing the poly-Ub chain after recognizing the target protein [21].

Before proteolysis, the target protein is unfolded by a hexameric ring composed of Rpt1 to Rpt6 subunits. Because this process consumes energy, each subunit acts as an ATPase. The unfolded polypeptide is mechanically pushed into the channel formed by the core particle to undergo lysis [22].

## 3. Proteasome Inhibitors

Inhibition of the UPS turned out to be a successful approach to cancer treatment, especially for hematological malignancies, MM and MCL. Currently, three drugs targeting UPS have been approved for therapy in these diseases: BTZ, CFZ, and IXZ [3]. All three share a common mechanism—they function as PIs.

### 3.1. Structure and Mechanism of Action

**Figure 3:**
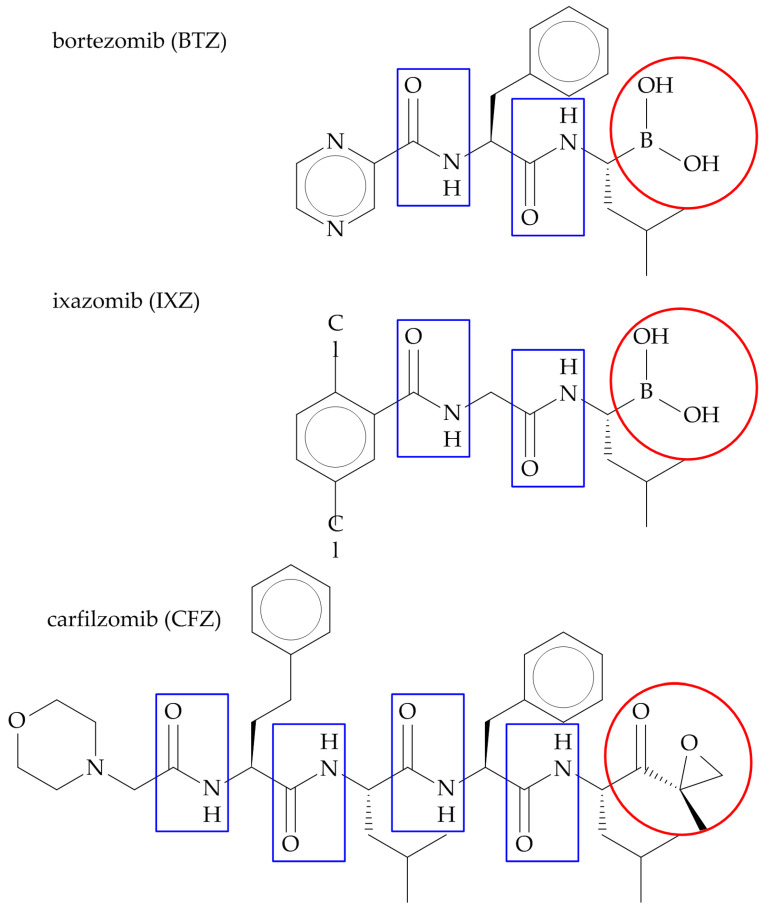
Chemical structures of bortezomib (BTZ), ixazomib (IXZ), and carfilzomib (CFZ). Red ellipses highlight the active groups: boronate in BTZ and IXZ and epoxyketone in CFZ. Blue rectangles indicate peptide bonds, indicating that all three compounds are oligopeptides.

The chemical structures of BTZ, CFZ, and IXZ are presented in Figure 3. These compounds are oligopeptides with an active boronate (BTZ and IXZ) or epoxyketone (CFZ) group [23]. They all selectively inhibit the proteasome β5 subunit activity, i.e., the chymotrypsin-like activity. BTZ and IXZ are reversible β5 inhibitors, whereas CFZ is irreversible [24].

These three PIs also inhibit the immunoproteasome β5i subunit. The lowest specificity characterizes BTZ, as it was shown to decrease the activity of other proteases, such as cathepsin A, cathepsin G, tripeptidyl peptidase II, and the mitochondrial protease LonP1. CFZ and IXZ appear to be more specific for proteasomes and do not interfere with the enzymes listed above [25].

The proteasome has a broad spectrum of substrates, which explains the complex impact of PIs on cells. The most evident mechanism involves the disruption of protein homeostasis. Under normal conditions, misfolded and damaged proteins are polyubiquitylated by E1, E2, and E3 enzymes and then degraded in the proteasome. After treatment with PIs, polyubiquitylated proteins accumulate in the cytosol after 20 min [26,27,28]. The proteasome is absent in the endoplasmic reticulum (ER). Thus, misfolded proteins are transported retrogradely to the cytosol for proteasomal degradation. Proteasome inhibition disrupts this transport, leading to ER stress and the dilation of ER cisternae [29,30,31]. The ER stress activates the unfolded protein response (UPR) pathway. UPR provides cells with a way to survive the ER stress, but when it is overloaded with misfolded proteins, it leads to cell death via apoptosis [32]. The ER stress was proved to increase the generation of reactive oxygen species (ROS) and to cause the activation of caspase 2 [33]. The proapoptotic NOXA protein is responsible for initiating apoptosis following ER stress, and its pivotal role in response to PIs was confirmed in several types of cancer [30,33].

ER stress is an essential mechanism for PI cytotoxicity, but it does not explain the selectivity of PIs for malignant cells. This selectivity is a consequence of distorting intracellular signal transduction pathways.

For instance, the nuclear factor κB (NF-κB) pathway is inhibited by PIs. NF-κB is a transcription factor that upregulates the expression of genes related to cell proliferation and survival, as well as proinflammatory and angiogenetic cytokines. NF-κB promotes malignant conversion and progression. Its activation requires lysis of the κB inhibitor (IκB). PIs inhibit the degradation of IκB, thus stabilizing this protein and preventing NF-κB activation [34,35].

PIs also impede cell cycle progression by stabilizing cell cycle inhibitors. For example, p21 and p27 are cyclin-dependent kinase (CDK) inhibitors. P21 can also arrest DNA synthesis through interaction with the proliferating cell nuclear antigen (PCNA). P21 and p27 arrest the cell cycle in the G1/S phase. In most cancers, these tumor suppressors are not entirely lost, but their localization and/or degradation are abnormal. Therefore, inhibiting the proteasome can lead to restoration of their function [36,37].

Despite the action of p21 and p27 in the G1/S phase, PIs were shown to arrest the cell cycle in the G2/M phase. As described above, PIs cause ER stress, leading to an elevated generation of ROS. It can induce DNA damage, which stabilizes p53. Blockage of p53 degradation results in its accumulation and arrests the cell cycle in the G2/M phase [36].

Another mechanism of action of PIs is sensitizing malignant cells to the immune system. The TNF-related apoptosis-inducing ligand (TRAIL) can serve as an example. TRAIL is a death receptor agonist, and its cytotoxic activity increases in the presence of PIs in several types of cancer [38]. PIs also enhance the antitumor activity of natural killer cells; similar results were obtained for dendritic cell-mediated immunity [30,39].

### 3.2. Resistance to Proteasome Inhibitors

Resistance of malignant cells to treatment is one of the most significant challenges in cancer therapy. PIs are no exception; cancerous cells can adapt to proteasome inhibition and develop resistance. BTZ resistance has been studied extensively, but many resistance mechanisms probably apply to other PIs.

It should also be noted that most research has been performed in vitro in cell culture models, and, therefore, it may not precisely reflect the conditions occurring in patients. Specific mechanisms of resistance, although consistently identified in different cell lines, have yet to be observed in clinical samples.

Mutations in the therapeutic target are one of the mechanisms of resistance. The β5 proteasome subunit is encoded by the *PSMB5* gene. Several point mutations in the *PSMB5* gene have been detected in multiple BTZ-resistant cell lines so far, and some of them decrease the BTZ affinity to the β5 subunit active site. These mutations do not always confer cross-resistance with other PIs [40]. Nevertheless, up to now, there has been only a single MM patient with confirmed *PSMB5* mutations contributing to the resistance [41].

Another common mechanism of resistance to anticancer agents is drug efflux. The multidrug resistance protein 1 (MDR1), also termed P-glycoprotein 1 (P-gp) or ATP-binding cassette sub-family B member 1 (ABCB1), is a membrane transporter responsible for removing xenobiotics from cells. In most cases, this protein is overexpressed by cancer cells, making them resistant to chemotherapeutics [42]. MDR1 preferentially transports hydrophobic, neutral, or positively charged molecules. Therefore, CFZ is known to be efficiently transported by MDR1, whereas BTZ and IXZ have a lower affinity to this pump due to their acidic boronate group [4,43].

The other mechanisms of PI resistance do not interfere with the interaction of PIs with the proteasome. They are adaptive mechanisms that mend the pathways disrupted by PIs and thus facilitate cell survival in the presence of PIs.

PIs inhibit protein degradation, leading to the accumulation of misfolded and damaged proteins. Consequently, cells utilize alternative protein-degradation pathways, including the aggresome–autophagy pathway [44]. Accumulating misfolded proteins expose hydrophobic residues and form insoluble aggregates. These aggregates are transported along microtubules to create aggresomes. This process requires histone deacetylase 6 (HDAC6), which binds the aggregates and the transporting protein, dynein [45]. The aggresomes then recruit autophagy machinery via sequestosome-1 (SQSTM1/p62), leading to autophagosome formation and, eventually, lysosomal degradation [46].

Malignant cells also tend to relieve the ER stress imposed by PIs. This can be achieved by overexpressing heat shock proteins (HSPs), also called chaperones, which assist in protein folding and are present in the ER. Two chaperones, glucose-regulated protein 78 (GRP78/BiP) and HSP90, are known to play a role in resistance to PIs [4]. PI-resistant cells can also adapt to oxidative stress by inducing an antioxidant response. This is mediated by increased levels of glutathione, glutathione-S-transferase, and other associated enzymes [47].

PIs arrest the cell cycle by inducing the accumulation of cell cycle inhibitors, such as p21, p27, and p53. These proteins inhibit cyclin-dependent kinases and halt cell proliferation. Malignant cells acquire resistance by removing the cell cycle inhibitors from the nucleus. Exportin-1 (XPO1) is the primary transporter responsible for the efflux of cell cycle inhibitors from the nucleus into the cytoplasm, and its overexpression is one of the mechanisms of PI resistance [6].

In patients, the resistance of cancer cells should be perceived in the context of the microenvironment, e.g., the extracellular matrix and neighboring non-malignant cells. The bone marrow microenvironment (BMME) was shown to participate in the resistance of MM cells to PIs. This effect is mediated by the secretion of different growth factors, cytokines, adhesion molecules, and exosomes, which modulate signal transduction pathways within the malignant cells [48]. For example, interleukin (IL)-6 prevents MM cell apoptosis and increases vascular endothelial growth factor (VEGF) production [49]. VEGF and IL-1β induce IL-6 expression, contributing to MM progression and resistance [50]. Adhesion of MM cells to the BMME cells via integrins also activates pro-survival pathways [51]. Exosomes and microRNAs also play a role in BMME-related PI resistance, resulting in a complex network of interactions [48].

## 4. The Strategies to Overcome Resistance to Proteasome Inhibitors

Over the last decade, much effort has been put into understanding how malignant cells adapt to PIs and acquire resistance to them. As new mechanisms of resistance are discovered (e.g., BMME involvement), new potential methods to overcome these mechanisms emerge. Several potential targets have been identified and tested, mostly in cell culture conditions. In this part of the article, we have aimed to review the research concerning attempts to overcome resistance to PIs. We briefly discuss agents and targets evaluated in preclinical models, but we mainly concentrate on drugs tested in clinical trials or approved as treatment options in humans.

**Figure 4:**
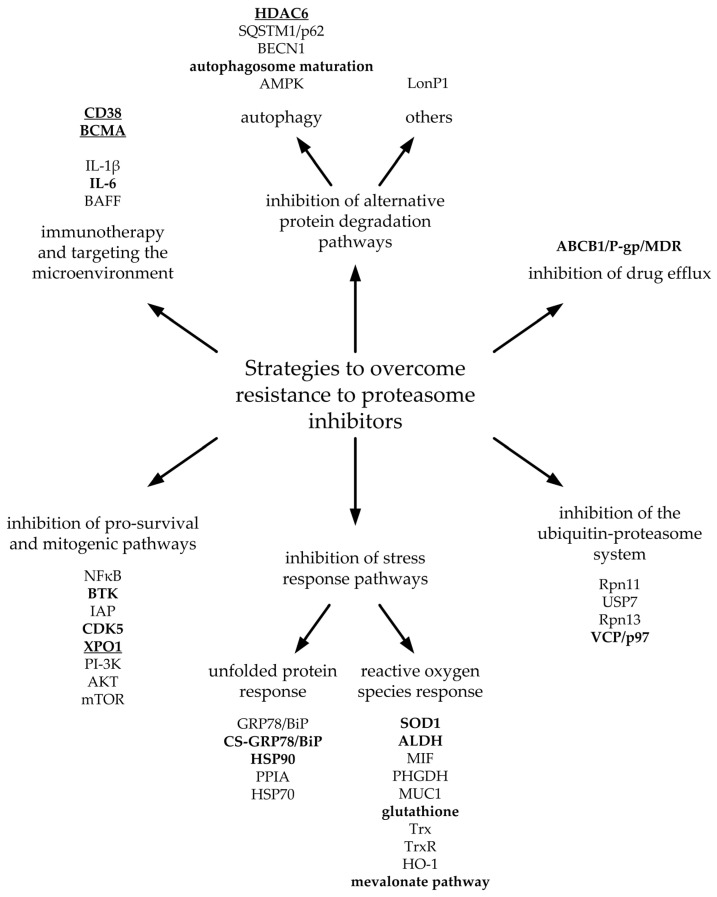
Diagram of potential targets for overcoming resistance to proteasome inhibitors. Bold font denotes targets that have been evaluated in clinical trials. Underlined targets have been approved by the FDA and EMA for treatment in combination with proteasome inhibitors.

The potential therapeutic targets are abundant, but different researchers have investigated other parts of the same pathway in many cases. We have decided to group these targets into strategies to clarify the subject. The potential targets described below are also presented in Figure 4.

### 4.1. Targeting the Ubiquitin–Proteasome System

The proteasome is the central component of the UPS, and it is also one of the targets in the work to overcome PI resistance. PIs target the β5 proteasome subunit. Therefore, mutations in the *PSMB5* gene encoding this subunit are a well-established mechanism of PI resistance. They affect the structure of the catalytic site and the substrate-binding pocket. Since the introduction of BTZ into clinical use, it has been shown that altering the BTZ structure can recover its affinity to the mutated β5 subunit. CFZ and IXZ, sometimes collectively called “second-generation” PIs, aimed to overcome this resistance mechanism. Indeed, it was confirmed in a clinical trial that CFZ exerts anticancer activity in BTZ-resistant MM patients [52], whereas the data for IXZ in BTZ-resistant patients are limited [53,54]. Regardless of the activity of second-generation PIs in BTZ-resistant patients, CFZ and IXZ are also characterized by a more favorable safety profile than BTZ, especially in the context of peripheral neuropathy [54].

More PIs are still being developed to overcome BTZ resistance and reduce adverse effects [23]. Marizomib, an irreversible inhibitor of all three proteasome catalytic subunits, entered the phase 2 clinical trial in MM patients and the phase 3 trial in glioblastoma patients [55,56]. Another example of PI in clinical trials is oprozomib, which demonstrated promising results in treating MM and Waldenström macroglobulinemia in the phase 1b/2 clinical trial [57]. Delanzomib was also investigated in a phase 1b/2 clinical trial, but its further development was discontinued due to insufficient efficacy [58]. New PIs will probably be introduced into clinical use, but they will likely share limitations with the current PIs. Patients will presumably develop resistance to the latest drugs as well because of their similar mechanism of action.

Several attempts to inhibit the UPS at other levels have been made so far. Rpn11 removes poly-Ub chains from degraded proteins. Its expression is inversely correlated with BTZ-treated MM patients’ overall survival, and it is considered a potential therapeutic target in MM patients [59]. Pharmacological inhibition of Rpn11 with o-phenanthroline overcomes BTZ resistance in MM cell lines and patient-derived cells [60]. The low specificity of o-phenanthroline to Rpn11 and its affinity to metals may limit its use.

Knockout and inhibition of another deubiquitinase, ubiquitin-specific protease 7 (USP7), also overcomes BTZ resistance in MM cells by stabilizing IκB and suppressing the NF-κB pathway. USP7 is known to remove the poly-Ub tag from proteins, protecting them from degradation, so the interplay between USP7 and IκB remains elusive [61].

Rpn13 acts as a poly-Ub receptor in the 19S regulatory particle, and its inhibition with RA190 was shown to circumvent PI resistance in MM cell lines and patient-derived cells. Du et al. proved that this effect is mediated by the downregulation of superoxide dismutase (SOD1), an ROS scavenger [62], but Rpn13 inhibition also increases anti-MM immunity [63].

Another potential therapeutic target is valosin-containing protein (VCP), also called p97. Its primary function is segregating ER contents and the translocation of misfolded proteins from the ER to the cytosol for proteasomal degradation [64,65]. This process is known as ER-associated degradation (ERAD). The first developed p97 inhibitor, eeyarestatin I, was shown to act synergistically with BTZ in killing cancer cells [66]. CB-5083 is a new-generation p97 inhibitor that entered two phase 1 clinical trials: (i) in subjects with advanced solid tumors (NCT02243917) and (ii) in subjects with lymphoid hematological malignancies (NCT02223598). Nevertheless, both studies were terminated due to off-target ophthalmological toxicity [67].

### 4.2. Targeting Drug Efflux

Malignant cells remove xenobiotics, including chemotherapeutics, using membrane transporters. This process is known as drug efflux and is mediated by a family of ATP-binding cassette (ABC) transporters. ABCB1, also known as P-gp or MDR1, is the most critical transporter in drug efflux [68]. An HIV protease inhibitor, nelfinavir, also inhibits ABCB1 and resensitizes resistant MM cells to CFZ [69]. Nelfinavir, in combination with BTZ and dexamethasone (DXM), has recently been evaluated in PI-resistant relapsed/refractory multiple myeloma (RRMM) patients in a phase 2 clinical trial. The objective response rate (ORR) in patients who received this combination was comparable to the ORR of PI-sensitive patients treated with BTZ and DXM. Thus, nelfinavir appears to overcome PI resistance in RRMM patients [70,71]. Considering the long clinical experience with nelfinavir and its good safety profile, it has a high potential to be introduced in clinics.

### 4.3. Targeting Alternative Protein-Degradation Pathways

PIs block the major cytosolic protein-degradation pathway. Therefore, cells adapt by upregulating alternative proteolysis mechanisms, such as the aggresome–autophagy pathway. This pathway relies on accumulating unwanted proteins in aggresomes followed by sequestering the aggresomes with double membranes, which results in the formation of autophagosomes. The maturation of autophagosomes is associated with acidification of their contents and lysis with lysosomal enzymes.

Histone deacetylase 6 (HDAC6) is pivotal in transporting proteins to aggresomes. Inhibiting HDAC6 has been a successful approach to overcoming PI resistance. A phase 3 clinical trial of panobinostat, a non-selective HDAC inhibitor, showed an extension of progression-free survival in MM patients treated with panobinostat, BTZ, and DXM in comparison to those treated with BTZ and DXM alone [72]. The FDA and EMA approved panobinostat for the treatment of RRMM in 2015.

More HDAC inhibitors are being developed, and several were evaluated in clinical trials: vorinostat, ricolinostat, quisinostat, romidepsin, and others [73,74]. Ricolinostat is especially promising because it is the first HDAC6-selective inhibitor and, therefore, features a favorable safety profile compared to non-selective HDAC inhibitors. In a phase 1/2 clinical trial, ricolinostat overcame resistance to BTZ in RRMM patients [75].

Sequestosome-1 (SQSTM1/p62) binds to polyubiquitylated proteins in aggresomes and the autophagy machinery, directing the proteins to lysis. In this way, it links UPS with autophagy. It is also associated with Nrf2, a transcription factor related to antioxidant response. SQSTM1 contributes to PI resistance, and its inhibition overcomes BTZ resistance in MM cells [76,77].

Beclin-1 (BECN1) participates in autophagosome formation by forming a complex with autophagy proteins. Xia et al. resensitized BTZ-resistant cells by silencing BECN1 with short hairpin RNA (shRNA) and obtained a similar result using chloroquine, an autophagy inhibitor [78]. Recently, a molecule disrupting the interaction between BECN1 and autophagy proteins has been discovered [79]. This compound has not yet been tested in MM cells.

Chloroquine (CQ) and hydroxychloroquine (HCQ) are antimalarial agents acting as potent autophagy inhibitors. They impede the fusion of lysosomes with autophagosomes, preventing lysis of the latter’s cargo. After promising results in cell lines, both drugs were tested in clinical trials in combination with PIs. In a phase 2 study, CQ combined with BTZ and cyclophosphamide overcame BTZ resistance in RRMM patients [80]. HCQ underwent two phase 1 clinical trials. In the first one, a combination of HCQ and BTZ showed an increased response rate in RRMM patients [81]. The results of the second trial, assessing the combination of HCQ, BTZ, and DXM in RRMM patients (NCT04163107), have not been published to date.

AMP-activated kinase (AMPK) is an enzyme involved in metabolic regulation within cells, and its activation by metformin is a commonly applied treatment of type 2 diabetes mellitus. The impact of metformin on autophagy and PI resistance has been extensively studied, but the results remain inconsistent. Jagannathan et al. reported that metformin suppresses GRP78-dependent autophagy in MM cells, exhibiting synergy with BTZ [82]. In contrast, Schlesser et al. obtained contrary results in MCL and lung carcinoma cells, i.e., metformin increased resistance to BTZ [83]. The different types of cancer and distinct concentrations of metformin within these studies might have caused this contradiction. Therefore, it is necessary to further examine the influence of metformin on PI resistance.

Aside from autophagy, cells possess other alternative proteolysis mechanisms. For instance, mitochondrial proteases can degrade cytoplasmic proteins, which might compensate for proteasome inhibition. Indeed, Maneix et al. discovered that the mitochondrial protease LonP1 contributes to CFZ resistance, and its inhibition exerts a synergistic cytotoxic effect with CFZ in MM cells. This mechanism does not apply to BTZ resistance because BTZ itself inhibits LonP1 [84].

### 4.4. Targeting Stress Response Pathways

As described above, PIs lead to ER stress and ROS generation. PI-resistant cells frequently adapt to stress by upregulating stress response pathways.

HSPs are chaperones—they assist misfolded proteins to recover their correct conformation. Through binding misfolded proteins, chaperones relieve ER stress. GRP78/BiP is one of those chaperones, and its inhibition with metformin increases the sensitivity of MM cells to BTZ [82]. Some malignant cells, including MM cells, expose GRP78 on their surface (noted then as CS-GRP78). The function of CS-GRP78 in cell biology is mostly unknown. It seems to regulate multiple signal transduction pathways [85]. Rasche et al. reported a PI-resistant MM patient who regained PI sensitivity after administration of an anti-CS-GRP78 monoclonal antibody PAT-SM6 [86]. A phase 1 clinical trial confirmed the safety of PAT-SM6 [87].

HSP90 inhibitors have been studied in the context of reversing PI resistance, and several compounds have entered clinical trials [88,89]. Promising results were obtained for tanespimycin in a phase 2 trial in MM patients [90], and, therefore, it was further evaluated in two phase 3 trials (NCT00546780 and NCT00514371). Nevertheless, the results from these two trials have not been reported yet, despite their completion in 2010. The last clinical trial of an HSP90 inhibitor in MM was finished in 2013. This strategy appears not to be investigated anymore.

Cyclophilin A (CypA) is an enzyme involved in protein folding, and it has been shown to reduce oxidative stress. Cohen et al. have recently identified CypA as crucial for PI resistance in RRMM patients. The authors sensitized patient-derived cells to CFZ with a CypA inhibitor, cyclosporin [91]. Cyclosporin, however, is a potent immunosuppressive agent and is generally contraindicated in patients with malignancies.

Research aiming to induce ER stress via other mechanisms is less advanced. VER-155008, an HSP70 inhibitor, acts synergistically with BTZ on MM cell lines [92] and inhibits MM growth in vivo in the mouse xenograft model [93]. Kim et al. proved that loperamide, an antidiarrheal drug, resensitizes colon cancer cells to BTZ by increasing ER stress [94]. However, these targets have not been investigated in a clinical setting.

Apart from HSPs, numerous other enzymes involved in response to ROS and oxidative stress contribute to PI resistance. ROS scavengers and related proteins offer many potential therapeutic targets.

Superoxide dismutase (SOD) inactivates superoxide radicals [95]. Disulfiram, an anti-alcoholism drug, acts as a copper and zinc chelator, thus interfering with SOD activity [96]. It appears that disulfiram can also overcome BTZ resistance both in acute myeloid leukemia and MM cell lines [97,98]. The anticancer activity of disulfiram is more complex because this compound also modulates other enzymes and pathways, such as aldehyde dehydrogenase, caspase-3, and the JNK pathway [99]. A phase 1 clinical trial assessing the efficacy of disulfiram and copper gluconate in MM patients was terminated recently without results (NCT04521335). Promising results were obtained in MM cells treated with a specific SOD1 inhibitor, LCS-1 [62]. Wang et al. resensitized MM cells to CFZ by inhibiting macrophage migration inhibitory factor (MIF), which acts as a chaperone for SOD1 and provides its proper folding [100]. These consistent results obtained by reducing SOD1 activity in different ways confirm its essential role in PI resistance.

Another crucial intracellular antioxidant is the tripeptide glutathione. A high glutathione concentration is associated with PI resistance; reducing its synthesis should recover sensitivity to PIs. Xuan et al. reported that phosphoglycerate dehydrogenase (PHGDH), an enzyme involved in the serine biosynthesis pathway, is also engaged in glutathione synthesis. PHGDH inhibition or knockout restores MM cells’ sensitivity to BTZ [101,102]. In malignant cells, glutathione synthesis is upregulated by a surface molecule: mucin-1 C-terminal subunit (MUC1-C). After Yin et al. had showed that MUC1-C inhibitor GO-203 overcomes BTZ resistance in MM cell lines [103], a phase 1 clinical trial was scheduled to assess the safety of GO-203 in combination with BTZ in MM patients (NCT02658396). The trial was not performed due to a lack of funding.

Recently, omega-3 fatty acids were reported to activate glutathione degradation in MM cells, and, therefore, they act synergistically with BTZ. However, the influence of these compounds on cell lines was inconsistent and depended on the treatment’s timing; the effect was only observed in cells treated with omega-3 fatty acids before exposure to BTZ. Omega-3 fatty acids have been studied as potential antineoplastic agents because their mechanism of action also includes inhibition of the NF-κB pathway. A phase 2 clinical trial was scheduled to assess whether omega-3 supplementation could prevent the progression of MM, chronic lymphocytic leukemia (CLL), and monoclonal gammopathy of undetermined significance (NCT00899353). The study has never been completed [104,105].

Thioredoxin (Trx) also functions as a redox regulator, which reduces oxidized proteins. Oxidized Trx is then reduced by thioredoxin reductase (TrxR), which transfers electrons from NADPH. Inhibition of either Trx or TrxR makes MM cells sensitive to PIs [106,107]. Auranofin, a TrxR inhibitor, is currently used to treat rheumatic arthritis, and it has undergone several clinical trials on patients suffering from different malignancies. Inhibition of TrxR leads to the induction of another redox protein, heme oxygenase-1 (HO-1). Hence, PI resistance in MM cells can be overcome by HO-1 inhibitors as well [108].

Another way to increase oxidative stress is by interfering with electron transport machinery in mitochondria. Coenzyme Q is an essential electron synthesized in the mevalonate pathway. Inhibitors of this pathway, such as statins, were proven to reduce mortality in MM patients [109,110]. Simvastatin appears to have the potential to overcome BTZ resistance in MM cells, and several clinical trials have been planned to prove it. The anti-MM effect of simvastatin was not observed in patients [111,112].

### 4.5. Targeting Survival and Cell Cycle Regulators

PIs exert their anticancer activity by modulating intracellular signal transduction pathways, many of which regulate survival, cell cycle, and death. Malignant cells adapt to this interference by adequately regulating these pathways. Additional disruption of these pathways is yet another strategy to circumvent PI resistance.

As mentioned above, the prosurvival NF-κB pathway is one of the main targets of PIs. Therefore, several studies on NF-κB inhibitors have been performed with hopes of overcoming PI resistance. Albendazole, an antihelminth drug, was found to inhibit the NF-κB pathway. Yi et al. showed that albendazole can restore MM cell sensitivity to BTZ in cell culture and mouse xenograft models [113].

A network of multiple upstream and downstream regulatory proteins regulates the NF-κB pathway. For instance, the Bruton kinase (BTK) is essential for NF-κB activation. Ibrutinib, a BTK inhibitor used in treating CLL, has been extensively examined for its ability to overcome PI resistance in MM. Several clinical trials were conducted, including two phase 2 trials investigating the combination of ibrutinib, DXM, and PIs in RRMM patients. The study investigating the combination of ibrutinib, DXM, and BTZ was terminated due to severe infections in treated patients [114]. In the other trial, patients received ibrutinib, DXM, and CFZ. The safety and efficacy of this combination were satisfactory [115].

Another NF-κB pathway regulator is the inhibitor of apoptosis protein (IAP). Zhou et al. showed that birinapant, an IAP inhibitor, exerted an anti-MM effect synergistically with BTZ. This was observed both in vitro and in vivo, even in BTZ-resistant cells [116]. Birinapant is a promising compound whose activity against various cancers was evaluated in several phase 2 clinical trials. It has not been approved for treatment of any cancer to date.

Cyclin-dependent kinase 5 (CDK5) is also engaged in NF-κB activation. Dinaciclib, a CDK5 inhibitor, presents a synergistic anticancer effect with BTZ in MM cells in vitro and in vivo. This agent, in combination with DXM and BTZ in RRMM patients, was studied in a phase 1 clinical trial (NCT01711528); we are still awaiting the results of the study. A phase 2 trial of dinaciclib as a single-agent treatment of MM gave promising results [117]. Apart from CDK5, dinaciclib has an affinity to CDK1, CDK2, and CDK9. Therefore, its activity can be partially due to direct cell cycle inhibition [118].

PIs arrest the cell cycle by preventing the degradation of cell cycle inhibitors, e.g., p21, p27, and p53. Cells circumvent this obstacle by removing these proteins from the nucleus into the cytoplasm, where they cannot fulfill their function. Exportin-1 (XPO1) is a crucial transporter responsible for this phenomenon. Therefore, its inhibition is a promising strategy to overcome PI resistance. Selinexor, an XPO1 inhibitor, was approved in 2019 for treating RRMM patients in combination with DXM, and a combination of selinexor, DXM, and BTZ was approved in 2020 [6]. The combination of selinexor with DXM and BTZ in MM was more effective than DXM and BTZ alone regarding outcome and fewer side effects [119]. However, the results of selinexor use in acute myeloid leukemia (AML) were not that promising. Selinexor did not improve AML patient survival. Moreover, selinexor-treated patients had an increased incidence of adverse events. The most common grade ≥ 3 adverse events were thrombocytopenia, febrile neutropenia, anemia, and hyponatremia [120]. More research is needed to determine the safety and effectiveness of selinexor and its combinations with other medications in different indications.

C-Myc is an oncogene that stimulates cell division and growth and inhibits apoptosis. It is regulated by the PI-3K/AKT/mTOR pathway, and several inhibitors of this pathway display synergistic activity with PIs. BKM120 and umbralisib are PI-3K inhibitors that resensitize MM cells to BTZ by downregulating c-Myc [121,122]. Umbralisib has been extensively studied in the treatment of lymphoma, and, in 2021, it was approved by the FDA for the treatment of marginal-zone lymphoma and follicular lymphoma. AKT inhibitors, such as TAS-117, also exhibit synergistic anti-MM activity with PIs [123]. Analogous results were obtained with other agents targeting the mTOR pathway, such as the antiasthmatic drug montelukast and the direct mTOR inhibitor rapamycin [124,125]. The consistent results obtained by inhibiting this pathway on various levels established its importance in overcoming PI resistance.

### 4.6. Targeting the Bone Marrow Microenvironment

The cells constituting the microenvironment in bone marrow (hematopoietic cells, immune cells, fibroblasts, and stromal cells) contribute to developing PI resistance. They generate pro-survival and mitogenic signals by expressing cytokines, adhesion molecules, growth factors, etc. The strategies to overcome this mechanism of PI resistance are less developed, mainly because the complex network of interactions cannot be investigated in a simple cell line monoculture.

Macrophages are a potential target in this setting. Proinflammatory macrophages infiltrate MM tumors and increase the stemness of cancerous cells. This effect depends on the macrophages’ secretion of interleukin-1β (IL-1β). Both IL-1β knockout and anakinra, an anti-IL-1β monoclonal antibody, abolished this process in mice [126].

Interleukin-6 (IL-6) was also shown to protect MM cells from apoptosis induced by steroids and chemotherapeutics, such as PIs. Blocking IL-6 signaling with monoclonal antibody siltuximab was effective in MM and improved the activity of BTZ and DXM in phase 1 and 2 clinical trials [127,128]. However, siltuximab failed to improve patient survival [129].

Another macrophage-derived molecule that increases PI resistance is the B-cell activating factor (BAFF). BAFF activates the pro-survival NF-κB, MAPK, and PI-3K/AKT pathways in MM cells. This resistance mechanism can be abolished by removing BAFF, either through genetic knockout or with an anti-BAFF antibody [130].

### 4.7. Immunotherapy

Protein homeostasis is the main sensitivity point of most cancer cells, which proliferate rapidly and rely on proper protein turnover. PIs are very effective, but once resistance mechanisms develop, they are not curative, and novel strategies for overcoming the resistance are urgently needed. One possible strategy in this field has focused on immune-based therapies that do not directly combat mechanisms of PI resistance but are nonetheless highly effective against PI-resistant cells [131]. Such indirect mechanisms include immunological elimination of resistant cells by targeting them with specific antibodies or CAR-T cells.

One example is daratumumab, which targets the CD38 antigen, which is highly expressed on the surface of myeloma cells. Daratumumab improved progression-free and overall survival in combination with PIs [132,133]. Similar effects were observed for isatuximab, an anti-CD38 monoclonal antibody [134].

Another immuno-approach targets the surface protein B cell maturation antigen (BCMA). In this context, the FDA approved two chimeric antigen receptors (CAR)-T cells and a bispecific T-cell-engaging antibody. This therapeutic strategy turned out to be highly effective, including PI-resistant patients, and improved survival [135,136,137].

Although immunotherapies provide promising results in overcoming PI resistance, they must be treated cautiously because cancers originating from the white blood cells may be too plastic and variable.

The therapeutic targets reviewed above are recapitulated in Table 1. 

### 4.8. Computational Approaches for the Discovery of Human Proteasome Inhibitors

With the constant development of in silico methods and the boost in the computational power of modern servers, the significance of theoretical approaches in drug research and development is undoubtedly increasing. Also, in the area of this review, major accomplishments have been achieved using molecular modeling methods. Those results have been carefully reviewed in the article by Guedes et al. [138].

This overview revised the application of multiple in silico methods, including quantum mechanics, molecular dynamics, pharmacophore generation, molecular docking, homology modeling, and virtual screening. These techniques identify and optimize new PIs and provide essential insights into the catalytic mechanisms and key interactions involved in proteasome inhibition. Several homology models were developed before the availability of the human proteasome crystallographic structure, which is necessary for structure-based methods, such as molecular docking.

A great deal of work was conducted using molecular docking calculations to support the significance of crucial interactions within the proteasome-binding pocket to determine structure–activity connections and to motivate synthetic attempts. Successful pharmacophore model generation enables the identification of key characteristics found in PIs. Based on prior research, structure-based and ligand-based virtual screening programs against chembridge, specs, and other databases yielded new and chemically diverse PIs. Furthermore, a few in silico techniques were coupled in a way that worked well together to explain and predict proteasome modulation.

In computational pharmacology, the knowledge about the structure of the studied macromolecule is usually the prerequisite for further research. X-ray crystallography has long been used to study the three-dimensional structure of proteasomes in various species, and the results have demonstrated that these structures are fundamentally similar. The crystallographic structure of the human constitutive 20S proteasome free and complexed with CFZ, however, was established for the first time by Harshbarger et al. [139]. Since then, with the availability of the crystalline structure of the human proteasome, the search for novel chemicals has advanced significantly and become even more fruitful. For example, in a recent work from 2024, Fernandes et al. [140] used molecular docking calculations to demonstrate that three mutations impacted the binding to the chymotrypsin-like active site. Specifically, these mutations, Ala49Thr, Ala50Val, and Cys52Phe, cause alterations in the S1 pocket, resulting in a modification of the interaction pattern between BTZ and the active site residues. The presence of larger side chains causes steric hindrance, resulting in reduced hydrogen bonds and interactions with essential residues. Consequently, BTZ can rotate and occupy a distinct location in the active site. This indicates a notable impact on the resistance mechanisms linked to the therapeutic use of BTZ. The acquired knowledge can direct the creation of more powerful and specific medications capable of overcoming resistance mechanisms similar to those shown with BTZ. This will ultimately improve the effectiveness of treatment for illnesses, such as MM and other cancers that use PIs.

## 5. Clinical Effects of Overcoming Resistance to Proteasome Inhibitors in Multiple Myeloma

The objective of all research regarding PI resistance is improving patients’ outcomes, understood not only as achieving better responses to treatment and lengthening survival but also as enhancing the quality of life. In the previous part of this review, we listed numerous potential therapeutic targets studied in preclinical and clinical settings. Here, we analyze the compounds investigated in phase 2 or 3 clinical trials, focusing on the reported efficacy and toxicity.

Phase 2 trials typically recruit a limited number of patients and are designed to assess the safety of an experimental agent. Evaluating efficacy in these studies is inaccurate because there is no control group, and only responses to treatment are reported. Because of the different patient characteristics, it is difficult to compare between various studies.

Phase 3 trials are generally superior to phase 2 trials in terms of a higher number of participants and the presence of a control group. They typically report not only treatment responses but also patients’ survival, which allows for calculating the hazard ratio (HR).

Response to treatment is evaluated according to the European Group for Blood and Marrow Transplantation (EBMT) criteria [141]. The objective response rate (ORR) is the percentage of patients with complete or partial response. This parameter estimates the number of patients who benefit from a given therapy.

Clinical trials of agents reviewed in this article are listed in Table 2.

Most agents were tested in patients with relapsed or refractory multiple myeloma (RRMM). These participants had received at least one line of treatment prior to enrollment in the studies, and their prognosis is generally worse than that of newly diagnosed patients.

As a single agent, oprozomib, a new PI, achieved 25–41% ORR in RRMM patients, depending on the dose. This moderate effect, comparable to older PIs, comes at the cost of significant toxicity, although oprozomib is less toxic than BTZ [57]. The drug efflux inhibitor nelfinavir, combined with BTZ and DXM, was associated with a higher ORR [70].

The histone deacetylase inhibitor panobinostat extended the median PFS by four months, which was sufficient for approval for RRMM treatment. The toxicity profile was similar to BTZ + DXM alone, but adverse effects were more frequent in the panobinostat group [72]. Vorinostat, another HDAC inhibitor, was also investigated in a phase 3 trial. This drug prolonged PFS by only one month, which was not clinically significant, and vorinostat did not gain approval [74]. Ricolinostat, an HDAC6-specific inhibitor, was evaluated in a phase 2 trial. The ORR was slightly lower than in the panobinostat trial. However, the safety profile of ricolinostat was better than that of panobinostat or vorinostat [75].

Chloroquine and two HSP90 inhibitors, tanespimycin and KW-2478, were evaluated in phase 2 clinical trials. The observed ORRs were 15–39%, and despite a favorable safety profile, the development of these compounds was halted [80,89,90]. Simvastatin was inefficient in MM patients, both in standard and high doses, and in both trials no objective responses were reported [111,112].

The BTK inhibitor ibrutinib achieved a promising ORR of 57–71% in two phase 2 trials in combination with different PIs and DXM. However, this was associated with significant toxicity. Many patients developed severe or fatal infections, raising safety concerns [114,115].

Selinexor, an XPO1 inhibitor, was approved for treating RRMM patients following the phase 3 clinical trial, which provided lengthening of the median PFS by five months and an increase in ORR. This was associated with an acceptable safety profile, with a higher frequency of some adverse events but a lower risk of peripheral neuropathy [119].

Anti-IL-6 antibodies, such as siltuximab, were expected to display synergy with PIs by targeting the microenvironment. This was not confirmed in clinical trials, as siltuximab failed to prolong PFS or increase ORR while increasing the risk of infections [129].

Immunotherapy turned out to be one of the most successful approaches to the treatment of RRMM patients. Anti-CD38 antibodies, which directly target MM cells, significantly prolong PFS, and daratumumab was shown to improve OS. These advantages come at the cost of increased risk of infections and cytopenias [133,134]. Anti-BCMA CAR-T cells and the bispecific antibody teclistamab are significant breakthroughs in RRMM therapy. They display excellent anti-MM activity [135,136,137]. However, the high incidence of cytokine release syndrome and severe neurotoxicity are limiting factors in their usage. Next-generation CAR-T cells, with improved safety mechanisms, will probably further revolutionize MM treatment [142].

## 6. Concluding Remarks and Future Perspectives

Over the last decade, significant progress has been made in understanding the mechanisms underlying PI resistance in malignant cells. In this review, we described over 40 different potential therapeutic targets whose inhibition could resensitize resistant cells to PI treatment.

Most of these targets were discovered in studies performed on cell line models. This is the simplest method for investigating sensitivity to treatment, but the results do not always translate to humans. The heterogeneity of MM cell lines also leads to inconsistent conclusions reached by different researchers. Moreover, it is impossible to investigate safety profiles in in vitro experiments. Some agents displayed promising activity in preclinical models, but their development was discontinued after clinical trials due to unacceptable adverse effects. This is especially visible in the strategy to target stress response pathways, where many clinical trials were terminated or withdrawn. The standard MM treatment of BTZ and immunomodulatory drugs already displays significant toxicity, and multiplying the adverse effects by introducing another agent might not be justified.

There are also no clear criteria for which cases of an interaction between PIs and an experimental drug can be considered “overcoming resistance”. The terms “synergistic effect”, “resensitization”, and “overcoming PI resistance” are often used interchangeably, although in some cases the study does not involve PI-resistant cells. It is possible that some of the drugs listed above do not revert PI resistance but their toxicity towards MM cells provides the observed effect.

On the other hand, the ultimate goal is improving patient outcomes, and whether a particular medication achieves this goal by reverting PI resistance or not is of secondary importance. For instance, immunotherapy is highly effective in RRMM patients, even though it was not designed to target specific mechanisms of PI resistance. The success of immunotherapy can be measured in the number of treatment regimens approved for clinical use, which is higher than all other strategies reviewed in this article combined.

Some experimental combinations listed in this article are highly likely to be introduced in clinical practice. This applies primarily to drugs that are already approved for the treatment of other diseases. For instance, nelfinavir, metformin, montelukast, disulfiram, and simvastatin are frequently used chronically. Their pharmacological properties, safety profile, and tolerability are well-established. This is an essential advantage over experimental drugs, which have not been widely used in patients, and their toxicity might be an important limiting factor.

Interestingly, 2024 is a double-jubilee year for studying PIs and the UPS. First, in May 2004, the first inhibitor BTZ was approved for human use, first by the FDA and then by the EMA. In the autumn of 2004, the Nobel Prize was awarded for discovering ubiquitin-dependent protein degradation.

The winners of the Nobel Prize in Chemistry are Avram Hershko, Aaron Ciehanover, and Irwin Rose [143]. But, in addition to their success, other scientists worked on the biology of the proteasome: Varshavsky, Wilk, Orłowski, Alfred Goldberg, Glickmann, Dahlman, Wojcik, Julian Adams, and many others [144,145,146,147,148,149].

Over time, interest in PIs has decreased. Although they are recognized medicines in MM, the therapeutic context of PIs still holds mysteries. One of the unknowns is the lack of clinical efficacy in treating solid tumors. Although they are effective in preclinical models, including the SCID model, several patient studies have been discontinued due to lack of effect or recurrence. Some proteasome inhibitors are of natural origin, e.g., lactacystin [150]. There is also a group of proteasome inhibitors isolated from marine organisms, mainly some invertebrates (soft corals and tunicates). The main proteasome inhibitor of marine origin is salinosporamide A (marizomib). Marizomib has fewer side effects than bortezomib. Marine compounds with proteasome-inhibiting activities exhibit different site-specific inhibition, which allows them to selectively inhibit different cleavage sites [151]. This property of this group of compounds gives hope for more selective activity and precise effects depending on the preferred site of action (constitutive proteasome, immunoproteasome, thymoproteasome, or spermatoproteasome).

Perhaps the mechanisms associated with the ubiquitination and deubiquitination of proteins described here are crucial for solid tumors’ resistance against PIs. We hope that future research focused on these mechanisms might enable the implementation of PIs in treating solid tumors as effective anticancer agents.

Moreover, the ability to selectively block, e.g., immunoproteasome, gives hope for new applications of these inhibitors in chronic inflammatory processes, such as rheumatoid arthritis, allergy, asthma, inflammatory bowel disease, etc. Many pharmaceuticals found novel, unexpected applications after years of clinical use, e.g., antimalarial agents in lupus erythematosus or thalidomide as an anticancer drug. Now, 20 years after introducing PIs to the clinic, is it time for new discoveries, implementations, and applications?

## Figures and Tables

**Table 1:** Summary of potential therapeutic targets for overcoming PI resistance.

NPL	Target	Inhibitor	Study Model	Remarks	Ref
**Targeting the ubiquitin–proteasome system**					
MM	Rpn11	o-phenanthroline	cell lines, patient-derived cells		[60]
MM	USP7	P5091, P22077	cell lines		[61]
MM	Rpn13	RA190	cell lines, patient-derived cells	RA190 also increases anti-MM immunity in vitro	[62,63]
MM	VCP/p97	eeyarestatin I, CB-5083	clinical trials (phase 1)	trials terminated due to off-target toxicity	[66,67]
**Targeting drug efflux**					
MM	ABCB1/P-gp/ MDR1	nelfinavir	clinical trials (phase 2)	nelfinavir is approved for treatment of HIV infection	[70,71]
**Targeting alternative protein degradation pathways**					
MM	HDAC	panobinostat	clinical trials (phase 3)	panobinostat is FDA- and EMA-approved for RRMM	[5,72]
MM	HDAC	vorinostat, romidepsin, quisinostat, etc.	clinical trials (phases 1–3)	vorinostat is FDA-approved for cutaneous T-cell lymphoma	[73,74]
MM	HDAC6	ricolinostat	clinical trials (phase 2)	ricolinostat has a better safety profile than non-selective HDAC inhibitors	[75]
MM	SQSTM1/p62	XRK3F2	cell lines		[76]
MM	BECN1	BECN1 shRNA	cell lines, patient-derived cells	new beclin-1 inhibitor was recently discovered	[78]
MM	autophagosome maturation	chloroquine	clinical trials (phase 2)	chloroquine is approved for treatment of malaria	[80]
MM	autophagosome maturation	hydroxychloroquine	clinical trials (phase 1)	hydroxychloroquine is approved for treatment of malaria	[81]
MM, MCL, NSCLC	AMPK	metformin	cell lines, patient-derived cells	inconsistent results: synergistic effect in MM, antagonistic in MCL; metformin is approved for treatment of type 2 diabetes mellitus	[82,83]
MM	LonP1	CDDO-Me	cell lines	not applicable to BTZ resistance	[84]
**Targeting stress response pathways**					
MM	GRP78/BiP	metformin	cell lines, patient-derived cells	metformin is approved for treatment of type 2 diabetes mellitus	[82]
MM	CS-GRP78/BiP	PAT-SM6 (IgM monoclonal antibody)	clinical trials (phase 1)	one patient regained BTZ sensitivity after PAT-SM6 administration	[86,87]
MM	HSP90	tanespimycin, NVP-AUY922, KW-2478, etc.	clinical trials (phases 1-3)	results of phase 3 trial of tanespimycin in RRMM patients not published	[88,89,90]
MM	CypA	cyclosporin A	patient-derived cells	cyclosporin A is an immunosuppressive agent, contraindicated in cancer patients	[91]
MM	HSP70	VER-155008	cell lines, xenograft model		[92,93]
CRC	ER stress	induction of ER stress with loperamide	cell lines	Loperamide is approved as an antidiarrheal drug	[94]
MM, AML	SOD1, ALDH	disulfiram	cell lines	a phase 1 clinical trial has been terminated; disulfiram is approved for treatment of alcoholism	[97,98]
MM	SOD1	LCS-1	cell lines, patient-derived cells		[62]
MM	MIF	4-IPP	cell lines		[100]
MM	PHGDH	NCT-503	cell lines, patient-derived cells		[102]
MM	MUC1	GO-203	cell lines	a phase 1 clinical trial was scheduled, but not conducted	[103]
MM	glutathione	omega-3 fatty acids	cell lines	a phase 2 clinical trial to assess if omega-3 could prevent MM, CLL, and MGUS progression was scheduled, but not completed	[104]
MM	Trx	PX12	cell lines		[107]
MM	TrxR	auranofin	cell lines	auranofin is approved for treatment of rheumatoid arthritis	[106]
MM	HO-1	ZnPPIX, LS1/71	cell lines		[108]
MM	mevalonate pathway	simvastatin	cell lines, clinical trials (phase 1/2)	simvastatin is approved for hypercholesterolemia	[110,111,112]
**Targeting survival and cell cycle regulators**					
MM	NF-κB pathway	albendazole	cell lines, xenograft model	albendazole is approved as an antihelminth drug	[113]
MM	BTK	ibrutinib	clinical trials (phase 2)	ibrutinib is approved for treatment of CLL	[114,115]
MM	IAP	birinapant	cell lines, xenograft model		[116]
MM	CDK5	dinaciclib	xenograft model, patient-derived cells	results of a phase 1 clinical trial for dinaciclib, BTZ, and DXM have not been published; phase 2 trial of single-agent dinaciclib brought positive results	[117,118]
MM	XPO1	selinexor	clinical trials (phase 3)	selinexor is FDA- and EMA-approved for RRMM	[6]
MM	PI-3K	BKM120, umbralisib	cell lines, xenograft model	umbralisib is approved for treatment of MZL and FL	[121,122]
MM	AKT	TAS-117	cell lines, xenograft model, patient-derived cells		[123]
MM	mTOR pathway	montelukast, rapamycin	cell lines, xenograft model	montelukast is approved as antiasthmatic drug	[124,125]
**Targeting the bone marrow microenvironment**					
MM	IL-1β	anakinra (monoclonal antibody)	mice	anakinra is approved as an immunosuppressive drug	[126]
MM	IL-6	siltuximab (monoclonal antibody)	clinical trials (phase 2)	siltuximab improved response to treatment, but not survival	[127,128,129]
MM	BAFF	monoclonal antibody	MM cell and macrophage co-culture		[130]
**Immunotherapy**					
MM	CD38	daratumumab, isatuximab (monoclonal antibodies)	clinical trials (phase 3)	both antibodies are FDA- and EMA-approved for RRMM	[132,133,134]
MM	BCMA	CAR-T cells; teclistamab (T-cell-engaging bispecific antibody)	clinical trials (phase 2)	CAR-T therapies and teclistamab are FDA- and EMA-approved for RRMM	[135,136,137]

AML—acute myeloid leukemia, CLL—chronic lymphocytic leukemia, CRC—colorectal carcinoma, FL—follicular lymphoma, MCL—mantle-cell lymphoma, MGUS—monoclonal gammopathy of undetermined significance, MM—multiple myeloma, MZL—marginal zone lymphoma, NPL—neoplasm, NSCLC—non-small-cell lung carcinoma, RRMM—refractory/relapsed multiple myeloma.

**Table 2:** Summary of clinical trials on overcoming PI resistance in multiple myeloma patients.

Phase	Population	Intervention	Control Group	Outcome	Toxicity	Ref
1b/2	102 pts RRMM	single-agent **oprozomib** **(new PI)** various dosing regimens	none	ORR: 41%, 28%, 25% (different doses)	gastrointestinal symptoms, fatigue, anemia, thrombocytopenia	[57]
2	34 pts PI-resistant MM	**nelfinavir (MDR1 inhibitor)** + BTZ + DXM	none	ORR: 65%	anemia, thrombocytopenia, infections, hyperglycemia	[70]
3	768 pts RRMM	**panobinostat (HDAC inhibitor)** + BTZ + DXM	*placebo* + BTZ + DXM	ORR: 61% vs. 55% PFS: 12 vs. 8 mos. HR: 0.63 (CI 0.52–0.76), *p* < 0.0001	gastrointestinal, fatigue, polyneuropathy; adverse events more common in the panobinostat group	[72]
3	637 pts RRMM	**vorinostat (HDAC inhibitor)** + BTZ	*placebo* + BTZ	ORR: 56% vs. 41% PFS: 8 vs. 7 mos. HR: 0.77 (CI 0.64–0.94), *p* = 0.01	adverse effects did not vary between the studied groups	[74]
1/2	116 pts RRMM	**ricolinostat (HDAC6 inhibitor)** + BTZ + DXM various dosing regimens	none	ORR: 29%, 37%, 25% (different doses)	renal failure, fatigue, anemia, diarrhea	[75]
2	11 pts RRMM	**chloroquine (autophagy inhibitor)** + BTZ + cyclophosphamide	none	ORR: 30%	fatigue, gastrointestinal symptoms, myalgia, polyneuropathy, cytopenias	[80]
1/2	72 pts RRMM	**tanespimycin (HSP90 inhibitor)** + BTZ	none	ORR: 15%	diarrhea, nausea, fatigue, thrombocytopenia, dizziness	[90]
1/2	80 pts RRMM	**KW-2478 (HSP90 inhibitor)** + BTZ	none	ORR: 39%	diarrhea, nausea, fatigue, vomiting	[89]
1/2	6 pts BTZ- or bendamustin-resistant MM	**simvastatin (mevalonate pathway inhibitor)** + BTZ or bendamustin	none	ORR: 0%	no drug-related toxicities observed	[111]
2	6 pts RRMM	single-agent high-dose **simvastatin (mevalonate pathway inhibitor)**	none	ORR: 0%	mild gastrointestinal symptoms, myalgia	[112]
2	76 pts RRMM	**ibrutinib (BTK inhibitor)** + BTZ + DXM	none	ORR: 57%	thrombocytopenia, gastrointestinal symptoms, fatigue, infections, polyneuropathy	[114]
1/2b	59 pts RRMM	**ibrutinib (BTK inhibitor)** + CFZ + DXM	none	ORR: 71%	thrombocytopenia, gastrointestinal symptoms, fatigue, cough, infections, hypertension	[115]
3	402 pts RRMM	**selinexor (XPO1 inhibitor)** + BTZ + DXM	*placebo* + BTZ + DXM	ORR: 77% vs. 62% PFS: 14 vs. 9 mos. HR: 0.70 (CI 0.53–0.93), *p* = 0.0075	thrombocytopenia, fatigue and anemia were more frequent in the selinexor group	[119]
2	106 pts newly diagnosed MM	**siltuximab (anti-IL-6 antibody)** + BTZ + melphalan + prednisone	*placebo* + BTZ + melphalan + prednisone	ORR: 88% vs. 80% PFS: 17 vs. 17 mos.	hematologic adverse events and infections were more common in the siltuximab group	[129]
3	498 pts RRMM	**daratumumab (anti-CD38 antibody)** + BTZ + DXM	*placebo* + BTZ + DXM	OS: 50 vs. 39 mos. HR: 0.74 (CI 0.59–0.92), *p* = 0.0075	cytopenias were more frequent in the daratumumab group	[133]
3	307 pts RRMM	**isatuximab (anti-CD38 antibody)** + pomalidomide + DXM	*placebo* + pomalidomide + DXM	ORR: 60% vs. 35% PFS: 12 vs. 7 mos. HR: 0.60 (CI 0.44–0.81), *p* = 0.001	infusion reactions, infections and diarrhea were more frequent in the isatuximab group	[134]
1/2	97 pts RRMM	**ciltacabtagene autoleucel (anti-BCMA CAR-T cells)**	none	ORR: 98% median OS and PFS not reached after 27.7 mos.	cytokine release syndrome, cytopenias, neurotoxicity	[135]
2	128 pts RRMM	**idecabtagene vicleucel** **(anti-BCMA CAR-T cells)**	none	ORR: 73% PFS: 9 mos.	cytokine release syndrome, cytopenias, neurotoxicity	[137]
1/2	165 pts RRMM	**teclistamab (anti-BCMA and anti-CD3 antibody)**	none	ORR: 63% PFS: 11 mos.	cytokine release syndrome, cytopenias, infections, neurotoxicity	[136]

BTZ—bortezomib, CFZ—carfilzomib, CI—95% confidence interval, DXM—dexamethasone, HR—hazard ratio, MM—multiple myeloma, mos.—months, ORR—objective response rate, PFS—progression-free survival, pts—patients, RRMM—relapsed or refractory multiple myeloma. Bold font denotes experimental agents evaluated in the trials.

## Data Availability

Not applicable.
